# Genetics, Diagnosis, and Management of Hürthle Cell Thyroid Neoplasms

**DOI:** 10.3389/fendo.2021.696386

**Published:** 2021-06-10

**Authors:** David G. McFadden, Peter M. Sadow

**Affiliations:** ^1^ Division of Endocrinology, Department of Internal Medicine, Department of Biochemistry, Program in Molecular Medicine, Harold C. Simmons Comprehensive Cancer Center, University of Texas Southwestern Medical Center, Dallas, TX, United States; ^2^ Departments of Pathology, Massachusetts General Hospital and Harvard Medical School, Boston, MA, United States

**Keywords:** thyroid, Hürthle cell, thyroid cancer, Hürthle cell carcinoma, oncocytic

## Abstract

Hürthle cell lesions have been a diagnostic conundrum in pathology since they were first recognized over a century ago. Controversy as to the name of the cell, the origin of the cell, and even which cells in particular may be designated as such still challenge pathologists and confound those treating patients with a diagnosis of “Hürthle cell” anything within the diagnosis, especially if that anything is a sizable mass lesion. The diagnosis of Hürthle cell adenoma (HCA) or Hürthle cell carcinoma (HCC) has typically relied on a judgement call by pathologists as to the presence or absence of capsular and/or vascular invasion of the adjacent thyroid parenchyma, easy to note in widely invasive disease and a somewhat subjective diagnosis for minimally invasive or borderline invasive disease. Diagnostic specificity, which has incorporated a sharp increase in molecular genetic studies of thyroid tumor subtypes and the integration of molecular testing into preoperative management protocols, continues to be challenged by Hürthle cell neoplasia. Here, we provide the improving yet still murky state of what is known about Hürthle cell tumor genetics, clinical management, and based upon what we are learning about the genetics of other thyroid tumors, how to manage expectations, by pathologists, clinicians, and patients, for more actionable, precise classifications of Hürthle cell tumors of the thyroid.

## History of the Hürthle Cell

**Figure 1:**
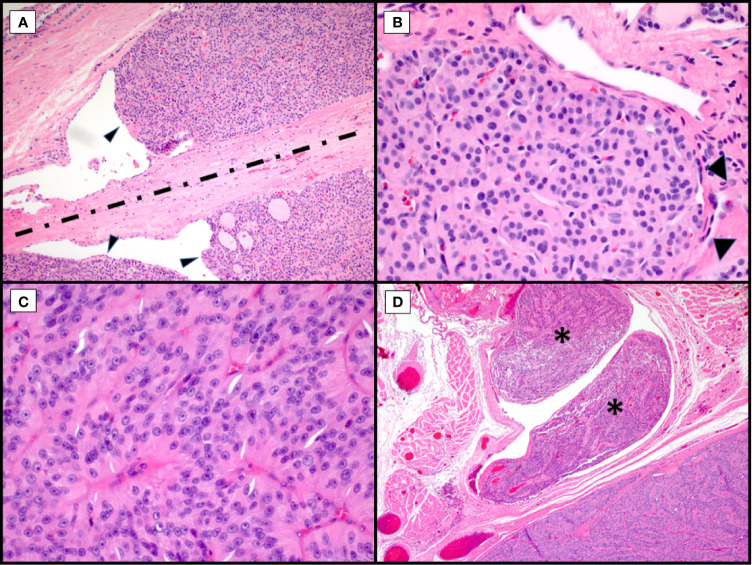
Morphological Controversies. Hematoxylin and eosin stains of Hürthle cell carcinoma. Endothelial wrapping or bulging of tumor into endothelial lined spaces raises the question of lymphatic invasion. Tumor bulging (**A**, arrowheads, 100X) is controversial with some requiring presence of intravascular thrombi. The band of fibrosis (**A**, discontinuous line) raises the question of biopsy site versus capsule with invasion with some discussion of hemosiderin deposition (not seen here) to indicate needle track. Tumor nodule with surrounding endothelial cells (**B**, arrowheads, 400X) in a space adjacent to the main tumor mass raises the possibility of capsular invasion versus vascular invasion versus focus of adjacent tumor in an unencapsulated lesion. Trabecular growth of true Hürthle cells (**C**, 400X) with round nuclei, pale nucleoplasm and prominent, centrally located nucleoli in Hürthle cell carcinoma. In the same tumor, with trabecular growth (**D**, 40X), shows an adjacent focus of overt vascular invasion in a large vessel located within the adjacent skeletal muscle (asterisks).

The Hürthle cell is of mysterious lineage. Thyroid-associated Hürthle cells are microscopically recognizable to pathologists with ease using a hematoxylin and eosin stain due to its abundant, lacy, oxyphilic cytoplasm with a large, round nucleus containing a prominent, typically centrally located nucleolus (**Figure 1**). Although deemed Hürthle cells when they are found within the thyroid gland, similar-appearing cells are seen in other sites, most commonly in salivary gland and the kidney. Indeed, other cell types, even hepatocytes, may resemble the Hürthle cells, with variable cellular architecture and less distinct nucleoli. An abundance of intracellular mitochondria of uncertain function underlies the dense, lacy cytoplasm apparent on histologic sections. Hürthle cells were first described by Max Askanazy, a Swiss-German pathologist, in 1898 (1), although the monicker was ascribed to Karl Hürthle, a German pathologist who actually identified parafollicular C cells in 1894. Despite this being corrected frequently in the literature over the last century, as having been described by Askanzazy, including a move toward designating these lesions as oncocytic, more descriptive than ascribed for discovery, suggested in 1950 by Herwig Hamperl, a German pathologist (2), the misnomer has taken hold, been emblazoned in the most recent edition of the WHO classification of endocrine tumors (3), and is widely used internationally.

## Hürthle Cell Neoplasia

Although the science is evolving, the exact origin of Hürthle cells in the thyroid is unclear, in terms of their physiologic function and from which progenitor they arise, a topic to be discussed elsewhere in this issue by Drs. Mete and Asa. Expression of thyroglobulin and other enzymes unique to the follicular epithelial cell of the thyroid in many Hürthle cell tumors suggests these cells evolve from the thyrocyte at some point in their development. However, this appears to take place prior to neoplastic transformation as mixed Hürthle cell/follicular lesions are not typically observed. Hürthle cell neoplasms have been an orphan disease group in the thyroid. To date, we have generally considered thyroid neoplasms to fall into one of two tumor types, follicular-derived thyroid tumors (FDTT, tumors of thyrocytes) or parafollicular tumors of c-cell/neural crest origin. Of the FDTT, we have adenomas and carcinomas and a myriad of described subtypes of each. However, of the follicular-derived (non-papillary) tumors, in the 2004 WHO, there were follicular adenomas (FA) (4) and follicular thyroid carcinomas (FTC) (5). There were no discrete chapters to describe Hürthle cell neoplasms (HCN) in this edition. HCN were described as unique oncocytic subcategories with the follicular adenoma and follicular thyroid carcinoma categories. So, although these tumor types were morphologically distinct lesions, for classification purposes, these tumors were lumped together with their follicular, non-papillary counterparts, with an additional, oncocytic variant of papillary thyroid carcinoma (not elaborated on here).

Behaviorally, however, this created a conundrum. Although listed as a subtype of follicular thyroid carcinoma, Hürthle cell carcinomas (HCC) were morphologically, behaviorally and genetically distinct, which we will discuss. Follicular thyroid carcinomas are well-known to metastasize in a manner unique to their architecture and morphology, avoiding lymphatic spread and localized neck disease and, instead, favoring more insidious metastatic patterns *via* a hematogenous routes to distant sites, including lung, bone, and brain (6, 7), whereas papillary thyroid carcinomas are more likely to present, in advanced cases, with localized neck disease with lymph node metastases (8). Hürthle cell carcinomas also follow a lesion-specific pattern of metastasis, employing the aggressive components of both disease subtypes, with advanced disease consisting of both lymph node metastasis to the central and lateral neck, along with vascular spread to distant tissue sites (9).

Regarding pathologic evaluation of these tumors, they are regarded as all follicular-patterned thyroid neoplasms. Unless overtly widely invasive malignancies, they are typically well-circumscribed, encapsulated or unencapsulated, and the diagnosis of malignancy involves invasion into the adjacent thyroid parenchyma or vascular invasion within or external to the lesional capsule (**Figure 1**) (3, 9, 10). Although Hürthle cell lesions in the thyroid can be unifocal, multinodular thyroids with a dominant Hürthle cell lesion or thyroids with a background chronic lymphocytic (Hashimoto) thyroiditis may have multiple Hürthle cell lesions or Hürthle cell change. This may confound the assessment for tumor invasion, especially in the setting of a dominant, thinly to unencapsulated lesion. Or, conversely, for thickly encapsulated Hürthle cell lesions with an abutting, unencapsulated Hürthle cell nodule, a decision needs to be made about transcapsular invasion versus an immediately adjacent, unrelated mass (**Figure 1A**). Levels may not reveal mushrooming invasion, and this then becomes a subjective assessment. For many pathologists, especially if the dominant mass is a large lesion, this becomes a challenging exercise self-restraint versus the risk of being incorrect; incorrect referring to both the biological truth and possibly review by an extrainstitutional pathologist with discordance in diagnosis.

**Figure 2:**
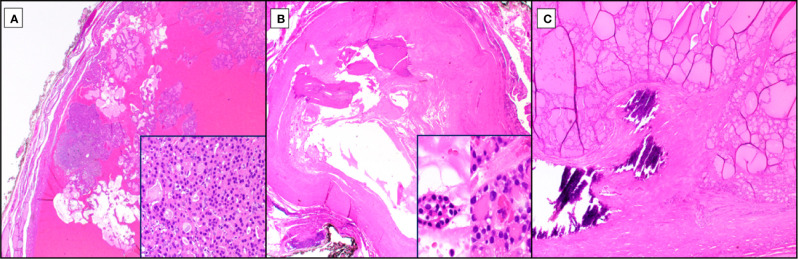
Reactive Changes in Hürthle Cell Neoplasia. Among the diverse reactive changes seen with Hürthle cell neoplasia (hematoxylin and eosin stain), especially post traumatic, are cystification (**A**, 20X magnification), ossification (**B**, 20X magnification), and capsular irregularity with coarse calcification (**C**, 40X magnification). Cystic changes due to hypoxia-related cellular drop out lead to papillary architectural formations at low power, lacking fibrovascular cores **(A)** but retaining Hürthle cell morphology and typically at least focal follicular architecture (**A**, inset, 1000X magnification). Exquisite hypoxia sensitivity can lead to complete ossification **(B)**, post-traumatic, of Hürthle cell tumors, occasionally with no residual cells or with rare clusters that may exhibit Hürthle cell features with associated nuclear atypia (**B**, inset, 1000X magnification). An additional feature, especially in tumors with thicker capsules, is intracapsular so-called “coarse” or “egg shell” calcifications which can distort the lesional growth pattern at the periphery and conferring a pseudoinvasive growth pattern **(C)**.

Trauma, especially with associated hemorrhage, can lead to rapid degenerative changes in Hürthle cell lesions, including cystification with papillary architecture, diffuse necrosis, hyalinization and ossification (**Figure 2**). These processes that may be triggered following needle biopsy or even palpation can lead to challenges in diagnosis and suggest an extreme sensitivity to tumor hypoxia or other consequences of vasculature interruption. Nuclei may lose their typical appearance and adopt nuclear membrane irregularities, including some clearing and loss of the prominent nucleolus for smaller chromocenters. Hürthle cells are known to have occasional intranuclear pseudoinclusions. Papillary cystic degenerative changes, plus or minus any of the other above changes could certainly lead to a (mis)diagnosis of papillary thyroid carcinoma (**Figure 2A**). Lack of fibrovascular cores or accompaniment by other features of traumatic/degenerative change, including necrosis, hemosiderin deposition or hyalinization should prompt a broader thought process and possibly trigger ancillary testing, including a BRAF immunohistochemical stain or molecular testing in particularly challenging/borderline cases. Additional hypoxia-related reactive changes can lead to nearly complete hyalinization or ossification of nodules, in some cases with osseous metaplasia with extramedullary hematopoiesis (**Figure 2B**). Small foci of residual Hürthle cells may be present (**Figure 2B**, insert), but they may have associated atypia that raise the possibility of oncocytic variant papillary thyroid carcinoma, and again, may trigger immunohistochemical/molecular work up depending upon the size and context of the lesion. Further, peripheral architectural abnormalities may be noted, in thickly encapsulated, variably densely encapsulated and thinly/unencapsulated lesions. For the former, thickly encapsulated or variably encapsulated lesions, calcifications may be present, so-called “coarse” or “egg shell” calcifications, with disrupted, pushing growth of the tumor into the capsule or intratumoral fibrosis giving the false impression of invasion (pseudoinvasion; **Figure 2C**). Care should be taken to not overinterpret lesions with fibrosis without overt invasion through the capsule into adjacent parenchyma, transcapsular invasion. And, even in cases with transcapsular invasion, if linear, consideration to fine needle aspiration site-related changes needs to be considered, especially if linear hemosiderin deposition is noted.

**Figure 3:**
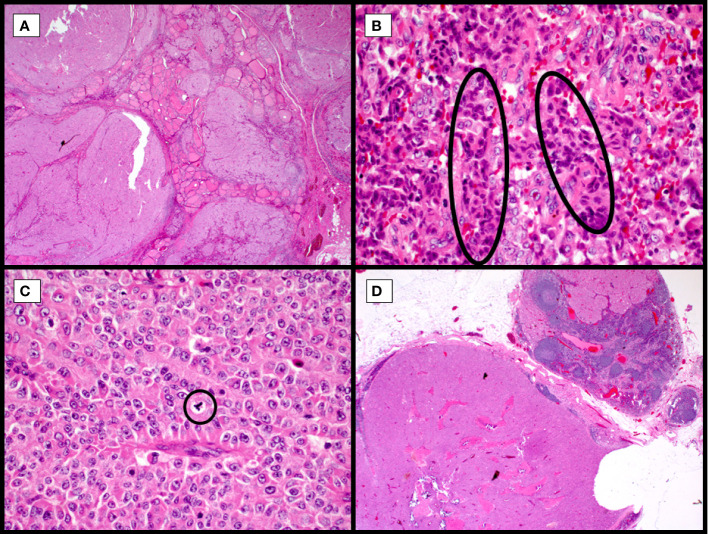
Widely invasive Hürthle cell carcinoma with high-grade features, hematoxylin and eosin stain. Multinodular growth of the Hurthle cell carcinoma into adjacent thyroid with no circumscription leads to non-controversial diagnosis of widely invasive Hurthle cell carcinoma (**A**, 40X). Collections of hyperchromatic, irregularly shaped nuclei with hypereosinophilic colloid, so-called burgeoning or incipient necrosis (**B**, circles, 400X). Frequent mitoses and scattered irregular (tripolar) mitoses (**C**, circle, 400X) and variably retained prominent nucleoli. Tumor metastasis to lateral neck lymph nodes (**D**, 20X), following a typical pattern of metastasis in Hürthle cell malignancies.

Widely invasive tumors are typically not a challenge, in terms of diagnosis, but there are other difficulties in their diagnosis (**Figure 3**). The presence of a solid, insular, or trabecular growth pattern, not infrequent in Hürthle cell tumors, raises the possibility for transformation to poorly differentiated thyroid carcinoma (PDTC; **Figure 3**). The original Turin criteria excluded oncocytic/Hürthle cell lesions (11), but this diagnostic line has become blurred more recently (12–15), a line that warrants molecular sorting rather than subjective histologic review, especially where outcomes and treatment modalities would be modified. These are often widely invasive tumors, with necrosis and mitotic activity, but while the cytoplasm may retain Hürthle cell features, the nuclei may be pleomorphic and lack the distinct prominent, centrally located nucleolus. Additionally, Hürthle cell tumors have variable growth patterns that can correspond with other follicular tumors, including macro and microfollicular lesions. However, being hypoxia sensitive, owing to the unique mitochondria-rich nature of these tumors, they are prone to cystic degeneration, papillary cystic architecture, hyalinization, ossification, and fibrosis.

## Preoperative Biopsy

Preoperative fine needle aspiration (FNA) biopsy is frequently performed for patients with solitary or multiple large thyroid nodules. A recent review by Jalaly and Baloch (9) shows a nice algorithmic approach to biopsied thyroid lesions with Hürthle cells present. Preoperative genetic testing is of some limited utility, although Hürthle cell neoplasia may be ruled in, ruled out, or deemed suspicious/neoplastic, depending upon assay choice, by molecular testing with indications for surgical excision (16, 17).

## Genetic Alterations in Hürthle Cell Neoplasms

**Figure 4:**
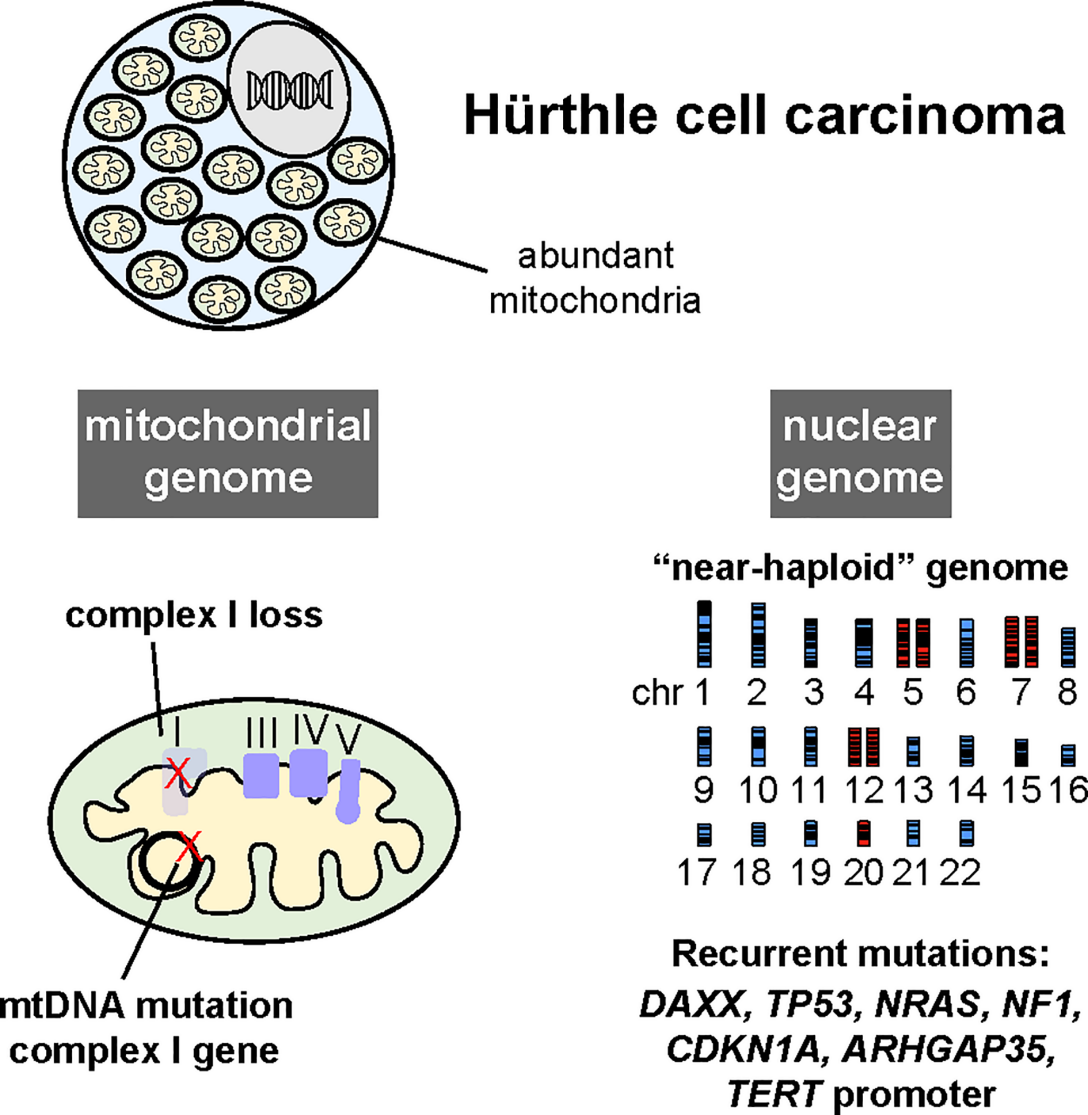
Hürthle cell carcinomas harbor recurrent mutations in genes encoding complex I of the electron transport chain in the mitochondrial genome (mtDNA), widespread loss of chromosomes leading to a near-haploid state, and recurrent mutations in several oncogenes and tumor suppressor genes, albeit at a low frequency. (Reproduced with permission from Gopal, RK, Kübler K, Calvo SE et al. Widespread chromosomal losses and mitochondrial DNA alterations as genetic drivers in Hürthle cell carcinoma. *Cancer Cell* 2018; 34:242-255.e5).

Several recent studies using next generation DNA sequencing methods have comprehensively characterized the somatic genomes of HCC. Cumulatively, these studies have built upon foundations established by prior focused studies of the nuclear and mitochondrial genomes to unveil unique features of the Hürthle cell neoplasm (HCN) somatic genome compared to other forms of neoplasia (summarized in **Figure 4**).

## Low Frequency of Canonical FDTC Driver Mutations

Several studies examined whether known thyroid oncogenes and tumor suppressor genes were recurrently mutated in HCC. Two studies reported recurrent RET-PTC translocations in HCCs using FISH and PCR methods (18, 19). However, in larger cohorts using DNA-sequencing based methods, RET-PTC (*CCDC6-RET and NCOA4-RET*) translocations were not detected, nor were RET-PTC translocations recurrently identified in molecular testing of FNA specimens with Hürthle cell cytology (20–26).

HCCs harbor mutations in known thyroid cancer oncogenes including *RAS*, *TSHR*, *EIF1AX*, *TP53*, *PTEN*, *BRAF*, *PAX8-PPARγ*, and *MEN1* (20, 22–31). However, mutations in these genes occur in a small fraction of HCC. Mutations in chromatin regulatory proteins were also reported at low frequency, similar to other clinically aggressive thyroid cancers (22, 24, 32, 33). Overall, these studies were consistent with the notion that HCC represented a molecularly distinct form of thyroid cancer.

## The Identification of Mitochondrial Genome Alterations in HCC

The accumulation of structurally abnormal mitochondria is a defining feature of HCN and other oncocytic neoplasms. The mitochondrial genome (mtDNA) is a small circular, bacterial-like genome that originated in the bacterial symbiont which evolved into the mitochondrion organelle. The mtDNA encodes a total of 37 genes, including 13 components of the mitochondrial electron transport chain (ETC). Deleterious germline mutations in the mtDNA, which are maternally inherited, result in variable impairment of the ETC and lead to an array of congenital diseases (34). In models of severe ETC impairment, morphologically abnormal mitochondria accumulate, which is thought to represent a compensatory response to impaired mitochondrial function (35).

Several groups have examined electron transport chain function and mtDNA in HCC and other thyroid cancers. A study of mtDNA mutations in a panel of thyroid cancers revealed frequent mtDNA mutations that clustered in genes encoding subunits of complex I of the ETC (36). A study of ATP levels and oxygen consumption in a panel of HCNs was also consistent with impaired ETC function (37). A key development in the field was the derivation of a cancer cell line from an HCC breast metastasis, XTC.UC1 (38). The XTC.UC1 cells were subsequently found to exhibit impaired ETC function and harbor disruptive mtDNA mutations in complex I and complex III (39).

Gasparre et al. then reported a complete mtDNA sequencing study of 45 oncocytic neoplasms (40). The majority of oncocytic tumors harbored mutations in the mtDNA that clustered within genes encoding subunits of complex I. Approximately one half (12/25) of the complex I mutations were predicted to be functionally disruptive (frameshift or nonsense mutations). This study established that mutations in mtDNA-encoded complex I subunits were highly recurrent in HCNs.

mtDNA exist at up to thousands of copies per cell. Therefore, mtDNA mutations can exist in a fraction (heteroplasmy) or all (homoplasmy) of the mtDNA copies. The relative fraction of deleterious mutants can impact the degree of ETC impairment, as well as phenotypes associated with individual mtDNA mutations (34). Gopal et al. extended analysis of mtDNA to a cohort of HCC that included sequentially resected primary and metastatic lesions from individual patients (24). In this study, mtDNA mutations were identified in 60% of HCC patients (24/40).

The depth of sequencing coverage provided by exome sequencing also enabled the investigators to estimate mtDNA heteroplasmy in the samples. According to pan-cancer sequencing data analyzed by Gopal et al., deleterious mutations in complex I subunits most frequently existed at low fraction across all cancer types (only a small fraction of mtDNA in the tumor carried the disruptive mutation) (41). This finding suggested that most tumors select against mutations that compromise complex I function. Interestingly, the opposite trend was observed in HCC: deleterious complex I mtDNA mutations existed in a very high fraction of mtDNA sequencing reads. This observation suggested that, unlike other forms of cancer, mutations that impaired complex I function were under positive selection in HCC and therefor possibly advantageous for tumor growth. Additionally, when the deleterious complex I mtDNA mutation was homoplasmic in the primary tumor, metastases derived from these primary tumors that maintained the mtDNA mutation at similar levels.

The results of this study supported the notion that loss of mitochondrial respiration, specifically through complex I impairment, was advantageous to HCC tumorigenesis, and might therefore act as a driver alteration in oncocytic neoplasms. Interestingly, similar findings were reported in renal oncocytoma which also harbored frequent mtDNA mutations in complex I subunits (42). These studies suggested that impairment of complex I function was an important early driver of oncocytic tumors arising in multiple tissues (common biology). Consistent with this notion, in a recent pan-cancer analysis of nuclear and mitochondrial genomes in over 2600 tumors, the combination of a non-silent mtDNA mutation without another known cancer driver gene mutation was most prevalent in thyroid and chromophobe kidney cancers (43).

## Chromosome Gains and Losses in HCC

Chromosomal aberrations are well established drivers of cancer initiation and progression. Early studies of overall DNA content in HCN suggested high frequency of aneuploidy (44). A study by Mazzuchelli et al. systemically assessed individual chromosomes using fluorescence situ hybridization (FISH) methods (45). Chromosome losses were observed more frequently compared to chromosomal gains in HCN. Specifically, monosomies of chromosomes 8, 22, and 2 were and gains of chromosome 7, 12, and 17 were observed. Another study using FISH reported also found that tumors with a greater number of chromosome losses exhibited poorer outcomes by retrospective analysis (46)(Erickson 2001).

Cover et al. utilized genome-wide DNA microarrays to more comprehensively evaluate chromosomal gains and losses in HCN (47). Whole chromosome losses were widespread leading to near haploidization of the genome in HCC. This finding was validated using single nucleus flow cytometry and FISH, and these alterations were unique in HCN compared to other thyroid cancers. Consistent with the earlier studies, Chr 7 was never haploid, and losses of Chrs 5, 12, 17, and 20 were infrequent.

Two recent exome sequencing studies of HCC reported similar findings and confirmed prior associations between widespread chromosome gains and losses and clinical outcomes. Ganly et al. reported that minimally invasive HCC were frequently diploid or harbored a few chromosomal losses or duplications. In contrast, widely invasive HCC more frequently harbored evidence of chromosomal amplifications, always involving chromosome 7 (22). Gopal et al. reported a high frequency of near-haploid HCC exhibiting widespread chromosomal losses (24). A subset of near-haploid HCC underwent whole genome duplication (WGD) that led to amplification of chromosome 7 and uniparental disomy of much of the genome. Interestingly, comparison of the ploidy state of related primary and metastatic tumors suggested that WGD was not a prerequisite for metastatic progression. Near-haploid primary tumors seeded metastases that generally maintained the near-haploid state. However, in a few patients, near-haploid primary tumors seeded metastases that exhibited WGD. Primary tumors that exhibited WGD also maintained this state in metastases.

The underlying basis of chromosomal loss remains unclear, although several hypotheses have been proposed (48, 49). Specific chromosomes appear to undergo gains or losses more frequently than others, suggesting that unique selective pressures are exerted on individual chromosomes. Chrs 1, 2, 3, 4, 6, 8, 9, 11, 14, 15, 21, 22 exhibited whole chromosome LOH in most cases reported by Gopal et al. In contrast, Chrs 5, 7, 12, and 20 exhibited increased copy number relative to other chromosomes. However, it is not clear whether duplication of Chrs 5, 7, 12, and 20 stemmed from positive selection for increased DNA copy number of these chromosomes, or rather originated because these Chrs rarely underwent chromosomal loss prior to WGD. Nonetheless, the fact that Chr 5, 7, 12, and 20 were infrequently lost suggested that genes encoded on these chromosomes contribute to HCC or growth or survival.

Together these studies suggest an advantage for progressive chromosomal losses during the outgrowth of HCC. Widespread chromosomal loss, as seen in HCC, is observed infrequently in cancer. However, cases of germ cell tumors, leukemia, renal oncocytoma, giant cell glioblastoma, mesothelioma, parathyroid and adrenocortical carcinoma harbor near-haploid genomes, typically in a much smaller fraction of tumors compared to HCC (42, 50–57). This observation suggests the near-haploid state can contribute to tumor growth, at least in some cancers.

## Clinical Challenges

HCCs are considered to exhibit a more aggressive clinical course compared to other forms of differentiated thyroid carcinoma (DTC), with higher incidence of distant metastases and more rapid progression of metastatic disease (58–61). However, it is important to note that aggressive clinical behavior is well-established primarily for patients presenting with widely invasive disease (extrathyroidal extension or extensive vascular invasion). Patients presenting with minimally invasive HCC exhibit excellent prognoses similar to FTC, based on available studies (61–64).

HCNs exhibit high ^18^fluoro-deoxy glucose (^18^FDG) uptake compared to other forms of thyroid cancer (65, 66). Conversely, HCCs are reported to exhibit less avidity for radioactive iodine (RAI), at least compared to FTC (60, 62, 67). Whether RAI has therapeutic benefit in HCC patients remains muddled. A retrospective analysis of the National Cancer Database (NCDB) suggested improved survival for HCC patients who receive RAI (68). However, disease specific survival and recurrence data were not available from the database. Another retrospective single institution chart review supported the use of RAI in HCC, but this study had notable limitations. First, there was no control group, and the HCC cases included in the study were largely minimally invasive cancers, which are likely to exhibit excellent outcomes regardless of intervention. The study was not able to discriminate between cervical neck RAI uptake from normal thyroid remnant or residual cancer, and no patients developed metastases in which uptake could be directly examined (69).

In contrast, data more clearly suggests that recurrent or metastatic HCC take up less RAI compared to other forms of DTC, which is logically consistent with the increased frequency of high-level FDG uptake in these cancers (62, 67). Overall, it remains questionable whether RAI has therapeutic benefit, especially in patients presenting with recurrent or metastatic disease.

A particular challenge for patients presenting with metastatic or recurrent disease is the general lack of actionable somatic mutations in HCC. Although actionable somatic mutations are occasionally detected, most HCC do not harbor mutations that rationally guide therapy. Therefore, cytotoxic chemotherapy, multikinase inhibition, and external beam radiation remain the primary options in the setting of recurrence of cancer progression in HCC.

## Summary and Future Directions

Hürthle cell neoplasia presents a challenge in both diagnosis and treatment. We are learning that HCN have unique molecular pathogenesis among FDTT, yet predicting biological behavior remains difficult, excluding widely invasive carcinomas. The increasing use of molecular testing during thyroid nodule diagnosis is an important avenue toward understanding the genetic underpinnings of the transition from adenoma to carcinoma as well as the development of specific therapies that target the underlying genetic drivers of these fascinating yet recalcitrant cancers.

## Author Contributions

Both authors contributed equally to the concept design, writing and editing of this manuscript. All authors contributed to the article and approved the submitted version.

## Funding

Support for this article has been provided, in part, by the Max Goodman Fund of the MGH Pathology Service. Support for PS is provided by a National Institutes of Health National Cancer Institute Grant 1P01CA240239-01.

## Conflict of Interest

The authors declare that the research was conducted in the absence of any commercial or financial relationships that could be construed as a potential conflict of interest.
